# Ways of Coping with Stress in Women Diagnosed with Breast Cancer: A Preliminary Study

**DOI:** 10.3390/healthcare13060609

**Published:** 2025-03-11

**Authors:** Agata Wypych-Ślusarska, Sandra Ociepka, Karolina Krupa-Kotara, Joanna Głogowska-Ligus, Klaudia Oleksiuk, Jerzy Słowiński, Antoniya Yanakieva

**Affiliations:** 1 Department of Epidemiology, Faculty of Public Health in Bytom, Medical University of Silesia in Katowice, 40-055 Katowice, Poland; kkrupa@sum.edu.pl (K.K.-K.); jglogowska@sum.edu.pl (J.G.-L.); koleksiuk@sum.edu.pl (K.O.); jslowinski@sum.edu.pl (J.S.); 2 Faculty of Public Health in Bytom, Medical University of Silesia in Katowice, 41-902 Bytom, Poland; s89377@365.sum.edu.pl; 3 Department of Health Technology Assessment, Faculty of Public Health, Medical University of Sofia, 1431 Sofia, Bulgaria; antoniya.yanakieva@googlemail.com

**Keywords:** breast cancer, coping strategies, stress

## Abstract

**Background:** Cancer diagnosis causes a range of different emotions. It is also a factor that causes feelings of severe stress. Coping with stress is individual and depends on the person’s nature, environment, and support they receive. **Aim:** This study aimed to assess how women diagnosed with breast cancer cope with stress caused by the disease. **Methods:** A total of 111 women diagnosed with breast cancer participated in the study. The questionnaires were distributed electronically using Google Forms in online forums and groups on social media. The survey consisted of two parts: the original questions and the Mini-COPE questionnaire. The relationships between stress-coping strategies and age, having children, marital status, and life satisfaction were tested. The Mann–Whitney U test, Kruskal–Wallis test, and Dunn’s post-hoc test with Bonferroni correction were used for the analyses (*p* < 0.05). **Results:** Of the surveyed women, 54.9% reported that the moment of diagnosis was the most stressful. Feelings of fear and anxiety accompanied 30.5% of the women, and 24.7% at the time of diagnosis could not provide information about the disease. The dominant strategies were seeking emotional support (mean 2.12 ± 0.56) and seeking instrumental support (mean 2.06 ± 0.48). Women in the older age group, married women, and women with children were most likely to adopt the strategy of turning to religion. **Conclusions:** The dominant strategies were seeking emotional and instrumental support. The strategy of turning to religion was used more often by older patients and patients with children.

## 1. Introduction

Breast cancer is the most common cancer in women worldwide. In 2022, this cancer was diagnosed in 2.3 million women and has contributed to 670,000 deaths worldwide [1]. According to the Global Cancer Observatory GLOBOCAN, the highest incidence rate of breast cancer in women in 2020 was in East Asia and South America, and the same was true for the mortality rate [2]. Breast cancer accounted for 13.3% of all new cancer cases diagnosed at the EU-27 in 2020. Therefore, it is the most common cancer [3,4]. The incidence and mortality rates of breast cancer have significantly changed worldwide over the past decade. According to GLOBOCAN data, the number of new breast cancer cases globally has increased from approximately 1.7 million in 2012 to approximately 2.3 million in 2022 [5]. The global age-standardized mortality rate for breast cancer has decreased from 13.0 per 100,000 in 2012 to 12.7 per 100,000 in 2022 [5]. The incidence trends in Europe are mainly upward, and many factors have contributed to this trend. In contrast, mortality data from Europe shows a declining trend. This is primarily due to the high effectiveness of treatment and detection of the disease at an early stage [3,4]. In Poland, the number of new cases in 2022 was 21,554 and the number of deaths was 6611 [6]. The age-standardized world (ASW) rates were 60.58/100,000 and 13.69/100,000, respectively. The number of new breast cancer cases has increased from approximately 17,000 in 2012 to over 24,000 in 2022 [5]. The age-standardized mortality rate has shown a slight decrease, reflecting improvements in treatment and early detection [5].

The number of cancer survivors has increased significantly [7]. The 5-year relative survival rate in Poland is 73% [8]. Data from across Europe on 5-year survival rates show considerably higher results, ranging from 79% in Croatia to 93% in Cyprus [9].

Breast cancer is also associated with several health and psychosocial burdens that are related to improved survival rates. These burdens include the physical challenges of undergoing treatments such as surgery, chemotherapy, and radiation, which can lead to fatigue, pain, and other side effects. Additionally, the emotional and psychological impact of a breast cancer diagnosis can be profound, often resulting in anxiety, depression, and stress. Social aspects, such as changes in family dynamics, work–life balance, and financial strain, further contribute to the overall burden [7,10]. Patients often struggle with the long-term effects of their disease, such as side effects of treatment; changes in daily activities; disruptions in social, family, and work roles; and uncertainty about cancer recurrence and death [7]. Anxiety, fear, and depression are common at the time of diagnosis and increase with disease progression and symptoms such as pain and fatigue. Stress usually increases with the intensity of treatment and can persist after treatment ends, affecting these individuals for months or years [10]. Despite these challenges, advancements in medical treatment and supportive care have significantly improved survival rates, allowing many individuals to manage these burdens more effectively and maintain a better quality of life [10].

A stressful situation triggers the activation of a coping process, which can focus on emotions or problems. The choice of coping method depends on the assessment of the situation and may lead to a change in the previous assessment [11]. The initial response to stress is adaptive and prepares the body to cope with challenges posed by internal or external environmental factors or stressors. However, if exposure to a stressor is perceived as intense, repeated, or prolonged, the stress response becomes maladaptive and detrimental to physiology and can cause harmful reactions, such as depression, anxiety, cognitive impairment, and heart disease [12]. Chronic stress can exacerbate pre-existing health problems and contribute to the development of destructive behaviors such as increased use of alcohol, tobacco, and other psychoactive substances.

Stress coping strategies constantly change cognitive and behavioral efforts to master specific external and internal demands judged by a person as burdening or exceeding their resources [13]. Talking about coping as a process takes into account the thoughts, emotional experiences, and intentional actions of an individual in a specific stressful situation [13].

The perception of cancer is an important element in coping with the stress it causes [14]. First, the patient feels the threat and fear of death, which is due to the general associations, stereotypes, and information that most societies have about cancer. Later, the image of the disease begins to be separated into a general image of cancer and an image of one’s illness. The disease can be interpreted as an evil that must be removed, from which one must escape, to which one must submit, or as a life task that must be met [14]. Patients can perceive their situation in many ways, some of which are more or less positive, and this can directly affect the acceptance process.

The goal of the coping process is to adapt to a disease and its treatment. There are two styles of coping with disease: constructive and destructive [15]. The constructive style is the perception of the disease as a task, a challenge that acts as a motivator, mobilizing the person to take action. The strategy of positive re-evaluation and a fighting spirit prevails here. By contrast, the destructive style is dominated by anxiety and resignation, which act as demotivators and discourage people from taking action. A significantly better prognosis, longer survival, and better quality of life are associated with active disease coping strategies. Active measures allow patients to better assess their capabilities, and encourage them to seek support and engage in social activities. A lack of acceptance of the disease can lead to negative emotions and destructive behavior. A person struggling with an illness that he/she has not accepted begins to experience difficulties in daily life. Exhaustion and feelings of helplessness have also been observed.

Patients with breast cancer face numerous physical and psychosocial challenges. While there have been significant advancements in medical treatment, psychosocial aspects, particularly stress management, remain underexplored. Despite the prevalence of stress among patients with breast cancer, research on effective coping strategies is limited. Most studies have focused on the general cancer population without addressing the unique needs of patients with breast cancer [16]. Existing studies have presented inconsistent findings regarding the effectiveness of different coping strategies. For instance, while some research highlights the benefits of emotional support and acceptance, others suggest that these strategies may not be universally effective [16,17].

The aim of this study was to fill these gaps by assessing the coping patterns of breast cancer patients, which may ultimately contribute to better psychosocial care and improved survival rates.

## 2. Materials and Methods

### 2.1. Research Tool

This study was conducted in the first half of 2024 in a group of 111 women diagnosed with breast cancer residing in Poland. The study was carried out using a survey questionnaire of our own authorship and the Inventory for Measuring Coping with Stress: Mini-COPE [18]. The author’s questionnaire included 23 questions related to socio-demographic data such as age and marital status, as well as questions about the type of breast cancer detected, the type of treatment used, or the time from diagnosis to treatment. Participants also indicated their reactions to information about cancer and their subjective feelings about the disease. It is important to note that the questionnaire was not validated because it primarily consisted of straightforward questions regarding demographic variables and disease course. The authors deemed that these questions did not require formal validation because of the descriptive and objective nature of the collected data.

The second part was the Mini-COPE standardized questionnaire developed by Zygfryd Juczynski and Nina Oginska-Bulik, which contains questions related to reactions to difficult or stressful life situations. The Mini-COPE scale is a tool used to survey healthy and ill adults [18]. It consists of 28 statements comprising 14 strategies (two assumptions for each coping style): active coping with stress, planning, positive re-evaluation, acceptance, humor, turning to religion, seeking emotional support, seeking instrumental support, substitute activities, denial, discharge, psychoactive substance use, cessation of activities, and blaming oneself. The aforementioned strategies make it possible to determine which strategies are most often used by subjects [18].

Each scale was evaluated separately by adding the scores for each of the two statements and then dividing the result of this sum by 2. The results obtained ranged from 0 to 3. The person completing the questionnaire chose several answers on a 4-degree scale: 0 = I almost never do this, 1= I rarely do this, 2 = I often do this, and 3 = I almost always do this. The scale contains four categories and corresponding strategies: active coping (active coping, planning, positive re-evaluation), helplessness (using psychoactive substances, stopping actions, blaming oneself), support-seeking (seeking emotional support, seeking instrumental support), and avoidant behavior (preoccupation with something else, denial, discharge).

### 2.2. Eligibility Criteria

The study used a purposive selection method: survey questionnaires were distributed on social media to groups of women diagnosed with breast cancer. The researchers employed a strategic selection process. Initially, they identified and targeted several large active Facebook groups dedicated to breast cancer support and advocacy. These groups were chosen based on their membership size, activity level, and diversity, in terms of demographics and geographic distribution. The researchers collaborated with group administrators to gain permission to distribute the survey. They ensured that the survey invitation was posted at different times and on different days to maximize reach and participation. Additionally, they included a brief introduction explaining the purpose of the study, ensuring transparency, and encouraging voluntary participation.

The primary criterion for inclusion in the study was a diagnosis of cancer (specifically breast cancer). The year of diagnosis, cancer stage, and treatment did not affect inclusion in the study. In addition to the diagnosis, the second inclusion criterion was consented to participate in the study, which was expressed by completing a questionnaire. Participation in this study was anonymous and voluntary. This study complied with the provisions of the Declaration of Helsinki. The study design, in light of the Act of 5 December 1996, on the professions of physician and dentist (Journal of Laws of 2011, No. 277, item 1634, as amended [19]), is not a medical experiment and does not require the approval of the Bioethics Committee of the Silesian Medical University in Katowice (opinion ID: PCN/CBN/0025/KB/079/23, 4 October 2023).

### 2.3. Statistical Analyses

The results were analyzed using Statistica 13.3, StatSoft Poland. Comparisons of the results of stress-coping strategies based on the Mini-COPE scale were made using the Friedman test and Dunn’s post-hoc test with Bonferroni correction. The relationship between stress coping strategies and age, having children, marital status, and life satisfaction was analyzed using the Mann–Whitney U test, Kruskal–Wallis test, and post-hoc Dunn’s test with Bonferroni correction. Dunn’s test with Bonferroni correction helps determine which groups differ from each other after finding an overall difference using the Kruskal–Wallis test. It can be applied after the Kruskal–Wallis test, which shows that at least one group is different from the others, but does not indicate which groups. Dunn’s test compares each pair of groups to check if they differ significantly, and the Bonferroni correction adjusts the significance level to account for multiple comparisons, reducing the risk of false positives. Statistical significance was set at *p* < 0.05.

## 3. Results

### 3.1. Study Group

The average age of the women surveyed was 45.6 ± 10.76 years; the youngest patient was 21 years old, and the oldest was 67. For statistical analyses, the participants were classified according to age groups: 21–44 years (n = 49; 44.1%) and 45–67 years (n = 62; 55.9%). Most women declared that they were married (64.9%) or single (24.3%). The remaining women were divorced (10.8%). The vast majority (70.3%) of the participants reported having children (Table 1).

### 3.2. Cancer

The youngest woman was 20 years old at the time of breast cancer diagnosis and the oldest was 65. The average age at diagnosis was 41.9 ± 12.23 years. The majority of diagnoses were malignant cancer, which accounted for 92.8% of diagnoses, whereas benign cancer accounted for 7.2% of cases. The most common forms of cancer treatment were chemotherapy (37.2%) and radiotherapy (18.2%). One in four women underwent mastectomy (25.1%) and 16.0% underwent surgical sparing treatment. The fewest number of women indicated hormone therapy (3.5%) as a type of cancer treatment. At the time of diagnosis, most women in the study group had to give up their work for some time. Only 36.9% of respondents remained economically active during their illness.

The respondents were asked whether the illness had changed their daily lives and activities. More than half of the women said that there was no change in their daily lives (63.1%), 31.5% needed help with daily chores, and 5.4% had to interrupt their studies due to illness.

**Figure 1:**
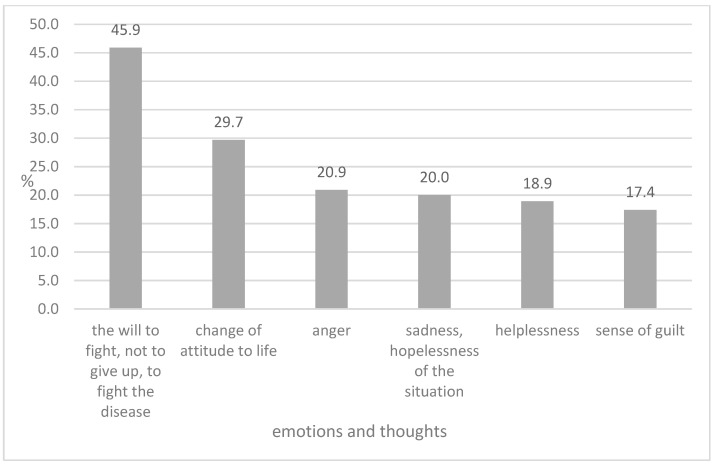
The categories of emotions and thoughts after breast cancer diagnosis. Percent of responses (percentages do not add up to 100 because it was a multiple choice question).

The respondents indicated various emotions and thoughts that accompanied them after hearing about the diagnosis (Figure 1).

Nearly half of the women surveyed declared a desire to fight the disease (45.9%) in the first days after hearing the diagnosis. The surveyed group also observed a change in their previous attitudes towards life (29.7%). The dominant emotions were anger (20.9%) and sadness (20.0%).

An important issue in cancer treatment is the support received. The women surveyed most often indicated family and loved ones (55.1%) as the most important source of support, followed by other patients (28.3%) and doctors (10.1%). They received the least support in their illness from a psycho-oncologist (6.5%).

The women also indicated the various activities they undertook to reduce the stress caused by the disease. These included habitual activities, such as walking (34.6%), talking to loved ones (26.9%), reading books and listening to audiobooks (18.5%), physical activity (13.0%), and listening to music (7.0%).

### 3.3. Strategies for Coping with Stress According to Mini-COPE

Descriptive statistics of the Mini-COPE scale related to stress-coping strategies of the study group are presented in Table 2.

The most frequently used strategies were seeking emotional support (2.12 ± 0.56) and seeking instrumental support (2.06 ± 0.48), as well as dealing with something else (1.95 ± 0.56). The least frequently used strategies were using psychoactive substances (0.27 ± 0.42) and employing a sense of humor (0.30 ± 0.42).

We investigated whether there was a significant difference in coping with stress according to women’s age, whether they had children, and their marital status (Table 3, Table 4 and Table 5).

The “turn to religion” strategy was statistically significantly more often used in the group of older women (45–67 years) than in younger women (21–44 years), as well as those with children. It was also observed that the strategies of “acceptance” and “sense of humor” were used slightly more often in the younger women group, but in this case, the differences between the groups did not prove statistically significant. The strategy of “denying” was more often undertaken in the group of women with children than among women without children, and the differences between the groups were on the verge of statistical significance.

Using the Kruskal–Wallis test, it was checked whether there was a difference between the independent variable (marital status) and the strategy taken (Table 5).

A statistically significant difference was noted between the turn to religion and marital status (*p* < 0.05). The other strategies of the Mini-COPE scale showed no relationship with the variable analyzed (marital status). To determine the variables that have a relationship, that is, which marital status is related to the strategy taken, the Dunn–Bonferroni post-hoc test was used. Analysis of the Dunn–Bonferroni post-hoc test showed that the strategy of turning to religion was more often undertaken by married women than by single women (*p* = 0.003).

## 4. Discussion

The main objective of this study was to assess the ways in which women diagnosed with breast cancer cope with stress caused by the disease.

Receiving a diagnosis of cancer is a difficult and stressful situation and often requires a redefinition of roles performed and adaptation to a new situation. In the survey conducted, almost one-third of the women needed help with daily chores, and for some, the disease meant having to stop studying. This is a revolution of sorts and a significant change to previous life, which can become a source of additional stress and affect mood and physical symptoms.

Managing stress during cancer treatment is of great clinical importance. Studies have indicated that chronic stress can significantly affect cancer treatment through a variety of biological mechanisms. Stress activates the hypothalamic-pituitary-adrenal axis (HPA) and sympathetic nervous system (SNS), which leads to the release of stress hormones such as cortisol, adrenaline, and noradrenaline [20]. Stress hormones can promote tumor growth and metastasis by increasing angiogenesis, which supplies tumors with nutrients and oxygen [20], and also increases the invasiveness of cancer cells, making it easier for them to spread to other parts of the body. Chronic stress also suppresses the immune system, reducing the ability of the body to detect and destroy cancer cells, which can lead to faster tumor progression and reduced effectiveness of immunotherapy. Furthermore, stress hormones can cause DNA damage and inhibit apoptosis, allowing cancer cells to survive and proliferate [20]. This resistance to apoptosis makes it difficult to eliminate cancer cells using treatments such as chemotherapy and radiotherapy [20].

News regarding the disease causes a range of different emotions, such as shock, disbelief, anger, depression, and fear. In the survey, the majority of reactions to the diagnosis were denial of the situation (33.9%) and feelings of anger and rage (20.9%). Denial is one of the most common reactions to disease diagnosis, particularly cancer. It is a defense mechanism that allows individuals to survive in the face of difficult news and adapt to new situations. Denial can have both positive and negative effects. Positive aspects include short-term anxiety and stress relief, which can help individuals cope with the initial trauma of diagnosis. However, long-term denial can hinder acceptance of the disease and negatively affect treatment. Studies indicate that diagnosis denial in cancer patients can affect between 4 and 47% of patients [21].

During illness, the support received is very important, as it can help patients get through the denial phase and better prepare for the next stages of treatment [22]. Our study showed that women received the most support from their family and loved ones (55.1%), followed by other cancer patients (28.3%). This observation suggests that good family relationships may translate into a sense of security. Support is very important for cancer patients, as it allows them to maintain a sense of self-worth. This feeling inspires the patient to have strength and fight the disease [23].

Stress is one of the factors that often accompanies a disease diagnosis, such as cancer, and it can affect the human body in both negative and positive ways. Hans Selye described stress as a physiological reaction of the body to the challenges that it faces. It can affect a person in either a mobilizing or, on the contrary, destructive manner, leading to many health problems [13]. The most common strategies in the study group were seeking emotional support and seeking instrumental support. These strategies involve seeking encouragement, understanding, and support from others, and seeking and receiving advice and help from others. In this study, more than half of the women indicated receiving support from loved ones, which is in line with the results of other studies on the most common strategies in a group of women with cancer [24]. The use of the aforementioned strategies may suggest that the support received may have been the result of women actively seeking such support or asking for help, but this hypothesis cannot be confirmed as the study did not ask who initiated the support.

Many studies of women diagnosed with breast cancer show that patients are most likely to use the strategies of active coping, turning to religion, and seeking emotional support [25,26,27,28,29]. The strategy of turning to religion refers to seeking comfort, guidance, or solutions through religious beliefs and practices, especially during times of stress, uncertainty, or crisis. This can involve activities such as prayer, attending religious services, reading sacred texts, or seeking counsel from religious leaders. People often turn to religion to find a sense of purpose, community, and hope, as well as to cope with life’s challenges and uncertainties.

The results of our research showed a relationship between age and coping strategy. This relationship was related to the strategy of turning to religion. Similar observations have also been reported in other studies [28,30,31]. For example, in an analysis of the impact of a breast cancer diagnosis on the religious/spiritual beliefs and practices of patients in the UK, after diagnosis, patients experienced a significant increase in belief in God, strength of faith, and private religious/spiritual practices [28]. Older patients were more likely to adopt religion as a strategy to cope with stress. In contrast, the results of a systematic review examining the impact of spiritual therapies on the quality of life of women with breast cancer showed that spiritual therapies have a positive impact on the quality of life of patients, especially in older age groups, who are more likely to use religion as a coping strategy [31]. The frequency of choosing this strategy among older women may be due to their period of religiosity. Between the ages of 40 and 60, a period of fulfilled religiosity occurs among religious people. This is the time of reaching religious maturity, which is characterized by strong faith [32,33].

Our study showed a correlation between having children and the strategy used. Women with children were more likely to choose the strategy of turning to religion. There was a similar correlation between the strategy adopted and women’s marital status. Married women were more likely to adopt the strategy of turning to religion than were unmarried women. Other studies suggest that religion can offer emotional support and a sense of meaning and purpose, which are particularly important for those with families [34,35]. People who have children and are married are more likely to use religion as a stress coping strategy. In addition, religious people are more likely to form a traditional family model, that is, get married, and are less likely to stay in informal relationships.

One way to reduce stress is to take up activities. The company’s own survey showed that most of the women engaged in activities to reduce stress, such as simple physical activity, but also talking to loved ones and reading books. Other studies have reported similar observations [36,37]. A meta-analysis analyzing the effect of physical activity on depression in patients with cancer showed that regular physical activity, especially aerobic activity, significantly reduced depressive symptoms and improved the quality of life of cancer patients. Physical activity also helps reduce anxiety and improve overall well-being [37,38]. Similar properties have been attributed to listening to one’s favorite music or reading books. A systematic review and meta-analysis examining the effects of music therapy on cancer patients showed that music therapy significantly reduced anxiety, depression, and pain, and improved patients’ quality of life [39,40]. Music therapy is particularly effective in reducing stress and improving mental wellbeing. Reading books is another way to improve concentration and reduce stress by turning thoughts to other areas unrelated to cancer [41].

The observation indicating the undertaking of active coping strategies to deal with stress may be a signal of good mental health for women diagnosed with breast cancer. The mechanisms of influence of the actions taken have been extensively described in literature, and their effectiveness has been proven.

Physical activity increases antioxidant levels in the body, which helps neutralize free radicals and reduce oxidative stress. Regulation of cortisol levels, in turn, leads to a reduction in anxiety and risk of depression. Regular physical activity strengthens the immune system, which can help fight cancer [42].

Studies have also indicated that music therapy has positive effects on mental health This is particularly relevant in patients with cancer. Music therapy contributes to a reduction in pro-inflammatory cytokines, resulting in reduced anxiety and improved mood [41]. Listening to music can also improve sleep quality, which is crucial for recovery and for stress reduction [43]. Reading books has a similar effect, which can be a temporary escape from daily problems and stress [40].

The results of our study indicate that breast cancer patients most often cope with stress by seeking emotional and instrumental support, which is consistent with the observations of Grajek et al. [44,45,46] regarding the importance of psychosocial factors in reducing stress in cancer patients. However, in contrast to the studies that focused on the impact of the COVID-19 pandemic on anxiety levels and coping strategies, our analyses show that regardless of external factors, women are more likely to rely on the support of loved ones, and older patients are more likely to choose to turn to religion. In addition, Grajek et al. [44,45,46] noted that avoidance and anxiety strategies were more common during the pandemic period, while active strategies predominated in our study, which may be due to differences in the stress context and specificity of the study group.

Many stress management techniques can also be helpful in cancer treatment, regardless of the location of the cancer (Table A1). However, no single recommended model exists for coping with stress. The strategies undertaken must be considered individually in each case in relation to the patient’s mental and physical abilities and stage of cancer advancement [47,48,49,50].

In a study by Kaleta-Pilarska [51], 56.5% of respondents did not accept their disease, and the level of acceptance was influenced by factors such as the presence of cancer in the family and satisfaction with medical care. These findings underscore the importance of social support and the quality of medical care in the process of adaptation to cancer, which is consistent with our observations on the role of emotional and instrumental support in coping with stress in breast cancer patients.

Although the results of the present study are in line with the observations of other authors, some limitations should be noted. First, a preliminary analysis of the results was conducted involving a rather small group of female patients. Therefore, it is possible that with a larger group of subjects, the responses could have been distributed differently, or relationships could have emerged that were not apparent at the level of the small group analysis. Moreover, the way in which the group was selected does not make it clear that the study is free of the “healthy respondent effect”. It is possible that patients with better physical and mental conditions were more likely to complete the survey. Thus, the results may have been distorted and may not reflect the true picture of the entire population. Therefore, the conclusions of this study were limited to the study group. Future research should consider changing the sampling method and focus on conducting surveys outside the online environment. The small sample size and the potential “healthy respondent effect” could lead to an overestimation of the effectiveness of certain coping strategies. In addition, the lack of longitudinal data limits the ability to assess the long-term impact of these strategies.

One of the main limitations of this study is that it was conducted online rather than in a real-world clinical environment. The lack of verification of participants’ identities in online groups can lead to uncertainty about the authenticity of the data. Conducting the study in clinics under controlled conditions could provide more reliable and valuable results, as participants would be directly monitored by medical staff.

The study protocol also did not include questions regarding the time elapsed since the diagnosis of breast cancer. Undoubtedly, this point should be included in a future study, as it will enable a full picture to be presented and the factors determining the stress-coping strategies undertaken by cancer patients. This study provides valuable insights into the coping strategies employed by breast cancer patients, particularly in the context of online support groups. This underscores the need for targeted interventions that address both the physical and psychosocial aspects of cancer care.

The findings of this study have significant policy implications, especially from the government’s perspective. Policies that support the development and implementation of comprehensive cancer care programs that include psychosocial support are needed. Governments should invest in training healthcare providers to recognize and address the psychosocial needs of patients with cancer. Additionally, funding should be allocated to support research on effective coping strategies and the development of resources for patients.

Future research should consider changing the sampling method and focusing on conducting the survey outside the online environment to avoid the “healthy respondent effect”. It should also include questions on the time elapsed since the diagnosis of breast cancer to provide a more comprehensive understanding of the factors influencing stress coping strategies. Longitudinal studies are needed to assess the long-term effectiveness of different coping strategies and identify any changes in coping mechanisms over time. Future studies should explore the impact of cultural and demographic factors on coping strategies to develop personalized interventions.

By addressing these limitations and expanding the scope of research, future studies can provide a more accurate and comprehensive understanding of stress management among patients with breast cancer, ultimately leading to better patient outcomes and improved quality of life.

The results of this study highlight the importance of addressing both physical and psychosocial aspects of breast cancer care. Healthcare providers should include psychosocial support as standard treatment for breast cancer. This includes offering counselling services, support groups, and stress management programs to help patients cope with the emotional and psychological burden of the disease. Developing personalized care plans that address each patient’s coping strategies and psychosocial needs can improve patient outcomes and quality of life. Conversely, implementing regular screening for mental disorders using validated tools, such as Mini-COPE, and early identification of stress can lead to timely intervention and better management of stress-related symptoms.

From the perspective of healthcare professionals, introducing education about the importance of psychosocial care and effective communication strategies could help build a supportive environment for patients and improve the patient–provider relationship.

Furthermore, the results of our study complement existing clinical practice guidelines for breast cancer, such as those provided by the European Society for Medical Oncology (ESMO) and the National Comprehensive Cancer Network (NCCN) [52,53]. These guidelines emphasize the importance of a multidisciplinary approach to breast cancer care, including psychosocial support and patient-centered care.

## 5. Conclusions

The most common methods used to reduce stress following breast cancer diagnosis are physical activity and talking to loved ones. Among the strategies used, the most common were seeking emotional support and seeking instrumental support. It was shown that patients who were married, had children, and were from an older age group were more likely to use the strategy of turning to religion. This study examined and highlighted the use of active strategies for coping with stress, which can be important in the process of recovery and remission of disease.

## Figures and Tables

**Table 1:** Characteristics of study group.

Category	Value
Number of women	111
Age (mean ± SD)	45.6 ± 10.76
Age category	
21–44 years	49 (44.14%)
45–67 years	62 (55.86%)
Marital status	
married	72 (64.9%)
single	24 (22.5%)
divorced	15 (10.8%)
Having children	78 (70.3%)
Age at breast cancer diagnosis (mean ± SD)	41.9 ± 12.23
Highest number of diagnoses at age	
46 years	9 (7.77%)
55 years	9 (7.77%)
56 years	9 (7.77%)
Type of cancer	
malignant	103 (92.8%)
benign	8 (7.2%)

**Table 2:** Gathered results of assessment for strategies for coping with stress according to the Mini-COPE scale in the study group.

Strategies for Coping with Stress	Arithmetic Mean	SD	Min.	Lower Quartile	Median	Upper Quartile	Max.
Active coping	1.29	0.78	0.00	1.50	1.00	1.50	3.00
Planning	1.12	0.69	0.00	1.50	1.00	1.50	3.00
Positive revaluation	1.22	0.63	0.00	1.50	1.00	1.50	3.00
Acceptance	1.60	0.55	0.50	2.00	1.50	2.00	3.00
Sense of humor	0.30	0.42	0.00	0.50	0.00	0.50	2.50
Turning to religion	1.07	0.80	0.00	2.00	1.00	2.00	3.00
Seeking emotional support	2.12	0.56	1.00	2.50	2.00	2.50	3.00
Seeking instrumental support	2.06	0.48	1.00	2.50	2.00	2.50	3.00
Dealing with something else	1.95	0.56	0.50	2.50	2.00	2.50	3.00
Denying	1.75	0.72	0.00	2.00	2.00	2.00	3.00
Discharge	1.55	0.48	0.50	2.00	1.50	2.00	3.00
Using psychoactive substances	0.27	0.42	0.00	0.50	0.00	0.50	1.50
Cessation of activities	0.89	0.52	0.00	1.00	1.00	1.00	2.50
Blaming yourself	1.22	0.59	0.00	1.50	1.00	1.50	2.50

**Table 3:** Age-specific strategies for coping with stress.

Strategies for Coping with Stress	Age	Arithmetic Mean	SD	Min.	Lower Quartile	Median	Upper Quartile	Max.	Mann–Whitney U Test
Active coping	1 2	1.42 1.19	0.85 0.71	0.00 0.00	1.00 1.00	1.00 1.00	2.00 1.50	3.00 3.00	Z = 1.19 *p* = 0.24
Planning	1 2	1.22 1.03	0.77 0.62	0.00 0.00	1.00 1.00	1.00 1.00	2.00 1.00	3.00 3.00	Z = 0.95 *p* = 0.37
Positive revaluation	1 2	1.20 1.23	0.67 0.61	0.00 0.00	1.00 1.00	1.00 1.00	1.50 1.50	3.00 3.00	Z = −0.02 *p* = 0.98
Acceptance	1 2	1.63 1.57	0.55 0.56	1.00 0.50	1.00 1.00	2.00 1.50	2.00 2.00	3.00 3.00	Z = 0.57 *p* = 0.57
Sense of humor	1 2	0.37 0.24	0.51 0.32	0.00 0.00	0.00 0.00	0.50 0.00	0.50 2.00	2.50 1.50	Z = 1.06 *p* = 0.29
Turning to religion	1 2	0.79 1.29	0.85 0.67	0.00 0.00	0.00 1.00	0.50 1.00	1.00 2.50	3.00 3.00	Z = −3.74 *p* < 0.001
Seeking emotional support	1 2	2.15 2.10	0.58 0.55	1.00 1.00	2.00 2.00	2.00 2.00	2.50 2.50	3.00 3.00	Z = 0.64 *p* = 0.52
Seeking instrumental support	1 2	2.01 2.10	0.51 0.44	1.00 1.00	2.00 2.00	2.00 2.00	2.00 2.50	3.00 3.00	Z = −1.05 *p* = 0.29
Dealing with something else	1 2	1.99 1.91	0.58 0.55	0.50 1.00	1.50 1.50	2.00 2.00	2.50 2.00	3.00 3.00	Z = 0.75 *p* = 0.46
Denying	1 2	1.66 1.81	0.81 0.65	0.00 1.00	1.00 1.50	1.50 2.00	2.00 2.00	3.00 3.00	Z = −1.01 *p* = 0.31
Discharge	1 2	1.55 1.56	0.49 0.47	0.50 0.50	1.50 1.00	1.50 1.50	2.00 2.00	2.50 3.00	Z = 0.20 *p* = 0.84
Using psychoactive substances	1 2	0.19 0.34	0.35 0.56	0.00 0.00	0.00 0.00	0.00 0.00	0.50 0.50	1.00 1.50	Z = −1.41 *p* = 0.16
Cessation of activities	1 2	0.85 0.93	0.52 0.52	0.00 0.00	0.50 0.50	1.00 1.00	1.00 1.00	2.00 2.50	Z = −0.75 *p* = 0.45
Blaming yourself	1 2	1.23 1.21	0.65 0.54	0.00 0.00	1.00 1.00	1.00 1.00	1.50 1.50	2.50 2.50	Z = 0.18 *p* = 0.86

1—Age 21–44 yr.; 2—Age 45–67 yr.

**Table 4:** Coping strategies for stress dependent on whether participants had children or not.

Strategies for Coping with Stress	Having Children	Arithmetic Mean	SD	Min.	Lower Quartile	Median	Upper Quartile	Max.	Mann–Whitney U Test
Active coping	1 2	1.23 1.44	0.71 0.92	0.00 0.00	1.00 1.00	1.00 1.00	1.50 2.00	3.00 3.00	Z = −1.04 *p* = 0.30
Planning	1 2	1.03 1.32	0.64 0.79	0.00 0.00	0.50 1.00	1.00 1.00	1.00 2.00	3.00 3.00	Z = −1.66 *p* = 0.10
Positive revaluation	1 2	1.20 1.26	0.63 0.66	0.00 0.00	1.00 1.00	1.00 1.00	1.50 1.50	3.00 3.00	Z = −0.55 *p* = 0.59
Acceptance	1 2	1.56 1.70	0.55 0.56	0.50 0.50	1.00 1.00	1.50 2.00	2.00 2.00	3.00 2.50	Z = −1.48 *p* = 0.14
Sense of humor	1 2	0.31 0.27	0.46 0.31	0.00 0.00	0.00 0.00	0.00 0.00	0.50 0.50	2.50 1.00	Z = −0.14 *p* = 0.89
Turning to religion	1 2	1.24 0.67	0.79 0.67	0.00 0.00	0.50 0.00	1.00 0.50	2.00 1.00	3.00 3.00	Z = 3.55 *p* < 0.001
Seeking emotional support	1 2	2.10 2.17	0.58 0.53	1.00 1.00	2.00 2.00	2.00 2.00	2.50 2.50	3.00 3.00	Z = −0.77 *p* = 0.44
Seeking instrumental support	1 2	2.07 2.03	0.46 0.51	1.00 1.00	2.00 2.00	2.00 2.00	2.50 2.50	3.00 3.00	Z = 0.34 *p* = 0.74
Dealing with something else	1 2	1.89 2.08	0.58 0.50	0.50 1.00	1.50 2.00	2.00 2.00	2.50 2.50	3.00 3.00	Z = −1.63 *p* = 0.10
Denying	1 2	1.83 1.55	0.67 0.81	0.50 0.00	1.50 1.00	2.00 1.00	2.00 2.00	3.00 3.00	Z = 1.94 *p* = 0.05
Discharge	1 2	1.53 1.61	0.49 0.45	0.50 0.50	1.00 1.50	1.50 1.50	2.00 2.00	3.00 2.50	Z = −0.85 *p* = 0.40
Using psychoactive substances	1 2	0.29 0.23	0.43 0.40	0.00 0.00	0.00 0.00	0.00 0.00	0.50 0.50	1.50 1.50	Z = 0.61 *p* = 0.54
Cessation of activities	1 2	0.91 0.85	0.46 0.64	0.00 0.00	0.50 0.50	1.00 1.00	1.00 1.00	2.00 2.50	Z = 0.68 *p* = 0.50
Blaming yourself	1 2	1.24 1.17	0.55 0.69	0.00 0.00	1.00 1.00	1.00 1.00	1.50 1.50	2.50 2.50	Z = 0.55 *p* = 0.58

1—having children; 2—not having children.

**Table 5:** Strategies for coping with stress in relation to marital status.

Strategies for Coping with Stress Active Coping	Marital Status	Arithmetic Mean	SD	Min.	Lower Quartile	Median	Upper Quartile	Max.	Mann–Whitney U Test	Dunn–Bonferroni Test		
										1	2	3
Active coping	1 2 3	1.32 1.25 1.28	0.84 0.72 0.75	0.00 0.00 0.00	1.00 1.00 1.00	1.00 1.00 1.00	2.00 1.50 1.50	3.00 2.50 3.00	H = 2.95 *p* = 0.40	1.00 1.00	1.00 1.00	1.00 1.00
Planning	1 2 3	1.18 0.92 1.10	0.76 0.63 0.66	0.00 0.00 0.00	0.50 0.50 1.00	1.00 1.00 1.00	2.00 1.00 1.50	3.00 2.00 3.00	H = 4.26 *p* = 0.23	1.00 1.00	1.00 1.00	1.00 1.00
Positive revaluation	1 2 3	1.16 1.08 1.26	0.66 0.51 0.66	0.00 0.00 0.00	1.00 1.00 1.00	1.00 1.00 1.00	1.50 1.50 1.75	3.00 2.00 3.00	H = 1.11 *p* = 0.77	1.00 1.00	1.00 1.00	1.00 1.00
Acceptance	1 2 3	1.66 1.50 1.60	0.61 0.48 0.55	0.50 1.00 0.50	1.00 1.00 1.00	2.00 1.50 1.50	2.00 2.00 2.00	2.50 2.00 3.00	H = 1.73 *p* = 0.63	1.00 1.00	1.00 1.00	1.00 1.00
Sense of humor	1 2 3	0.32 0.25 0.31	0.28 0.58 0.43	0.00 0.00 0.00	0.00 0.00 0.00	0.50 0.00 0.00	0.50 0.25 0.50	1.00 2.00 2.50	H = 3.49 *p* = 0.32	0.88 1.00	0.88 1.00	1.00 100
Turning to religion	1 2 3	0.56 1.13 1.19	0.49 1.03 0.75	0.00 0.00 0.00	0.00 0.25 0.50	0.50 0.75 1.00	1.00 2.00 2.00	1.50 3.00 3.00	H = 15.68 *p* = 0.001	0.45 0.003	0.45 1.00	0.003 1.00
Seeking emotional support	1 2 3	2.08 2.17 2.13	0.54 0.58 0.56	1.00 1.00 1.00	2.00 2.00 2.00	2.00 2.00 2.00	2.50 2.50 2.50	3.00 3.00 3.00	H = 2.36 *p* = 0.50	1.00 1.00	1.00 1.00	1.00 1.00
Seeking instrumental support	1 2 3	1.98 2.08 2.07	0.49 0.42 0.48	1.00 1.50 1.00	2.00 2.00 2.00	2.00 2.00 2.00	2.00 2.25 2.50	3.00 3.00 3.00	H = 3.36 *p* = 0.34	1.00 1.00	1.00 1.00	1.00 1.00
Dealing with something else	1 2 3	2.04 2.04 1.90	0.54 0.50 0.56	1.00 1.50 0.50	2.00 1.50 1.50	2.00 2.00 2.00	2.50 2.50 2.50	3.00 3.00 3.00	H = 4.22 *p* = 0.24	1.00 1.00	1.00 1.00	1.00 1.00
Denying	1 2 3	1.68 1.58 1.83	0.71 0.56 0.73	1.00 1.00 0.50	1.00 1.00 1.00	1.50 1.75 2.00	2.00 2.00 2.50	3.00 2.50 3.00	H = 4.78 *p* = 0.19	1.00 1.00	1.00 1.00	1.00 1.00
Discharge	1 2 3	1.58 1.42 1.56	0.43 0.42 0.50	0.50 0.50 0.50	1.50 1.25 1.00	1.50 1.50 1.50	2.00 1.50 2.00	2.00 2.00 3.00	H = 4.00 *p* = 0.26	1.00 1.00	1.00 1.00	1.00 1.00
Using psychoactive substances	1 2 3	0.18 0.08 0.35	0.38 0.29 0.44	0.00 0.00 0.00	0.00 0.00 0.00	0.00 0.00 0.00	0.00 0.00 0.75	1.50 1.00 1.50	H = 7.40 *p* = 0.06	1.00 0.77	1.00 0.37	0.77 0.37
Cessation of activities	1 2 3	0.84 0.92 0.90	0.62 0.42 0.50	0.00 0.00 0.00	0.50 0.75 0.50	1.00 1.00 1.00	1.00 1.00 1.00	2.50 1.50 2.00	H = 0.55 *p* = 0.91	1.00 1.00	1.00 1.00	1.00 1.00
Blaming yourself	1 2 3	1.24 1.04 1.26	0.69 0.78 0.50	0.00 0.00 0.50	1.00 0.25 1.00	1.00 1.00 1.00	1.50 1.75 1.50	2.50 2.00 2.50	H = 3.75 *p* = 0.29	1.00 1.00	1.00 1.00	1.00 1.00

1—Miss; 2—Divorced; 3—Married.

## Data Availability

Data are contained within the article.

## Appendix A

**Table A1:** Stress reduction techniques (own study based on [47,48,49,50]).

Technique	Description	Benefits
Cognitive-Behavioral Therapy (CBT)	A type of psychotherapy that helps patients identify and change negative thoughts and behaviors.	Reduces anxiety, depression, and stress; improves coping skills and overall quality of life.
Mindfulness-Based Stress Reduction (MBSR)	A program that incorporates mindfulness meditation to help patients focus on the present moment.	Reduces stress, anxiety, and depression; enhances emotional regulation and overall well-being.
Support Groups	Group meetings where patients can share experiences and receive emotional support from peers.	Provides emotional support, reduces feelings of isolation, and improves coping strategies.
Exercise	Physical activities such as walking, yoga, or tai chi, tailored to the patient’s abilities.	Improves physical health, reduces fatigue, and enhances mood and overall quality of life.
Relaxation Techniques	Techniques such as deep breathing, progressive muscle relaxation, and guided imagery.	Reduces stress and anxiety, promotes relaxation, and improves sleep quality.
Art Therapy	Creative activities such as drawing, painting, or music therapy to express emotions.	Provides a non-verbal outlet for emotions, reduces stress, and enhances emotional well-being.
Educational Interventions	Providing patients with information about their disease, treatment options, and coping strategies.	Reduces uncertainty, empowers patients, and improves adherence to treatment and overall outcomes.
